# Turnover of Lecanoroid Mycobionts and Their *Trebouxia* Photobionts Along an Elevation Gradient in Bolivia Highlights the Role of Environment in Structuring the Lichen Symbiosis

**DOI:** 10.3389/fmicb.2021.774839

**Published:** 2021-12-20

**Authors:** Ian D. Medeiros, Edyta Mazur, Jolanta Miadlikowska, Adam Flakus, Pamela Rodriguez-Flakus, Carlos J. Pardo-De la Hoz, Elżbieta Cieślak, Lucyna Śliwa, François Lutzoni

**Affiliations:** ^1^Department of Biology, Duke University, Durham, NC, United States; ^2^W. Szafer Institute of Botany, Polish Academy of Sciences (PAS), Kraków, Poland

**Keywords:** elevation gradients, systematics, symbiosis, Andes mountains, new PCR primer, Lecanoromycetes, Trebouxiophyceae, lichen biogeography

## Abstract

Shifts in climate along elevation gradients structure mycobiont–photobiont associations in lichens. We obtained mycobiont (lecanoroid Lecanoraceae) and photobiont (*Trebouxia* alga) DNA sequences from 89 lichen thalli collected in Bolivia from a ca. 4,700 m elevation gradient encompassing diverse natural communities and environmental conditions. The molecular dataset included six mycobiont loci (ITS, nrLSU, mtSSU, *RPB1*, *RPB2*, and *MCM7*) and two photobiont loci (ITS, *rbc*L); we designed new primers to amplify Lecanoraceae *RPB1* and *RPB2* with a nested PCR approach. Mycobionts belonged to *Lecanora* s.lat., *Bryonora*, *Myriolecis*, *Protoparmeliopsis*, the “*Lecanora*” *polytropa* group, and the “*L*.” saligna group. All of these clades except for *Lecanora* s.lat. occurred only at high elevation. No single species of Lecanoraceae was present along the entire elevation gradient, and individual clades were restricted to a subset of the gradient. Most Lecanoraceae samples represent species which have not previously been sequenced. *Trebouxia* clade C, which has not previously been recorded in association with species of Lecanoraceae, predominates at low- to mid-elevation sites. Photobionts from *Trebouxia* clade I occur at the upper extent of mid-elevation forest and at some open, high-elevation sites, while *Trebouxia* clades A and S dominate open habitats at high elevation. We did not find *Trebouxia* clade D. Several putative new species were found in *Trebouxia* clades A, C, and I. These included one putative species in clade A associated with *Myriolecis* species growing on limestone at high elevation and a novel lineage sister to the rest of clade C associated with *Lecanora* on bark in low-elevation grassland. Three different kinds of photobiont switching were observed, with certain mycobiont species associating with *Trebouxia* from different major clades, species within a major clade, or haplotypes within a species. Lecanoraceae mycobionts and *Trebouxia* photobionts exhibit species turnover along the elevation gradient, but with each partner having a different elevation threshold at which the community shifts completely. A phylogenetically defined sampling of a single diverse family of lichen-forming fungi may be sufficient to document regional patterns of *Trebouxia* diversity and distribution.

## Introduction

Alexander von Humboldt’s *Essai sur La Géographie des Plantes* was revolutionary for biogeography (Schrodt et al., 2019), but, with respect to lichens, Humboldt was limited by the nascent state of lichenology in the early 19th century. Drawing on observations from Europe and South America, Humboldt described lichens as “independent of the influence of the climates” (von Humboldt and Bonpland, 2009)—a statement that, with a modern understanding of lichen systematics and symbiosis, we now know is far from the truth. Climate does matter for lichens. There is turnover in lichenized fungi along elevation gradients, including those in the northern Andes (Wolf, 1993; Soto-Medina et al., 2019) that inspired Humboldt’s work. Furthermore, the mycobiont–photobiont interaction that defines the lichen symbiosis is affected by environmental conditions at micro and macro scales (e.g., James and Henssen, 1976; Peksa and Škaloud, 2011; Singh et al., 2017). What appears externally as the same lichen may actually represent a mycobiont species occurring with different photobiont species at different points in its range (Werth and Sork, 2014; Dal Grande et al., 2018).

Lichen photobiont biodiversity and patterns in the mycobiont–photobiont association have been most commonly studied at a regional, intra-biome scale at which climate is largely consistent [Hestmark et al., 2016; Jüriado et al., 2019; Yahr et al., 2004; but see Lu et al. (2018) for an intra-biome study along a climatic gradient] or at a global scale, at which climate may vary widely but regional patterns may not be apparent (Muggia et al., 2014; Nyati et al., 2014; Lutsak et al., 2016; Magain et al., 2017, 2018; Vančurová et al., 2018; Chagnon et al., 2019). Less commonly, regional photobiont turnover across adjacent biomes has been studied along gradients of latitude (Werth and Sork, 2014) or altitude (Dal Grande et al., 2018). In most studies, investigations of lichen photobionts have been structured around a specific mycobiont taxon, whether that taxon is a species (e.g., Blaha et al., 2006; Werth and Sork, 2014), genus (e.g., Vargas Castillo and Beck, 2012; Magain et al., 2017, 2018), or family (e.g., Helms, 2003; Nyati et al., 2014).

Lecanoraceae is one of the three largest families of lichenized fungi that associate with the green alga *Trebouxia*, the most common genus of lichen photobionts (Miadlikowska et al., 2006; Lücking et al., 2017; Muggia et al., 2018). The family is cosmopolitan and occurs on a range of substrates, typically with a crustose growth form (Brodo et al., 2001). Despite the family’s global distribution and diversity of corticolous species, the literature on the Lecanoraceae–*Trebouxia* association has mostly dealt with mycobiont taxa that are saxicolous, phylogenetically outside *Lecanora* s.str., and from Europe, North America, or Antarctica (Blaha et al., 2006; Guzow-Krzemińska, 2006; Pérez-Ortega et al., 2012; Ruprecht et al., 2012, 2020; Leavitt et al., 2015, 2016; Wagner et al., 2020). Photobiont studies that have included corticolous species of Lecanoraceae have generally done so in the context of sampling photobionts from across the lichen community of a small geographic area in the northern hemisphere (e.g., Singh et al., 2019).

*Trebouxia* is divided into five major clades: A, C, D, I, and S (Muggia et al., 2020; Xu et al., 2020). Clade D was recently identified and is known from Iceland, Svalbard, and Tierra del Fuego (Xu et al., 2020). Clade C is largely tropical, with a few temperate representatives. The remaining clades, A, I, and S, are cosmopolitan, including Antarctica. At the level of these major clades, lineages are distinguished by differences in pyrenoid structure (Muggia et al., 2020). The species-level classification of *Trebouxia* is still incompletely understood. Leavitt et al. (2015, 2016) and Muggia et al. (2020) implemented an alphanumeric classification system for putative *Trebouxia* species based on a so-called “barcode gap” species delimitation method (Puillandre et al., 2012). Although some of the species recognized by Muggia et al. (2020) correspond to named taxa, the majority have not been formally described.

In this paper, we investigate mycobiont–photobiont interactions in Lecanoraceae lichens from across an elevation gradient in Bolivia. Recent studies on lichen photobionts in Bolivia have uncovered putative new species of *Trentepohlia* and *Asterochloris* (Kosecka et al., 2020, 2021a), but the diversity of *Trebouxia* photobionts in Bolivia remains largely unexplored outside of one global study on photobionts of *Cetraria aculeata* (Lutsak et al., 2016). Our dataset includes corticolous and saxicolous species from *Lecanora* and other genera in Lecanoraceae and encompasses nearly 5,000 m of elevation change in the Andes mountains, south of where Humboldt developed his ideas on biogeography. We use this dataset to ask three main questions: (1) How is the biodiversity of Bolivian Lecanoraceae distributed, both in the phylogeny of this fungal family and along the elevation gradient? (2) What major clades and species of *Trebouxia* are associated with Lecanoraceae in Bolivia? (3) How do climate, substrate, and mycobiont host structure photobiont communities?

## Materials and Methods

### Field Sampling

**FIGURE 1:**
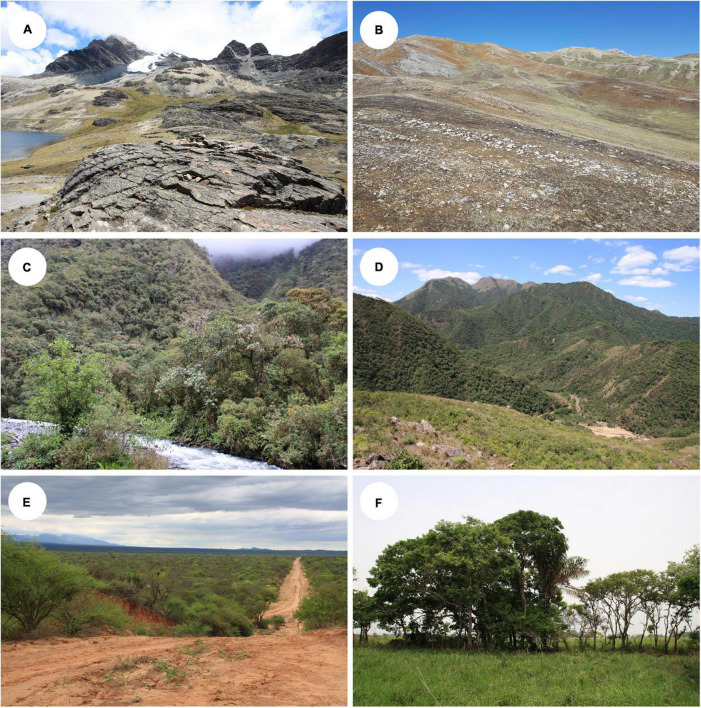
Habitats where Lecanoraceae lichens were collected. **(A)** La Paz, Bautista Saavedra, 14°48’9”S, 69°10’51”W, 4850 m, open high Andean vegetation with siliceous rocks. **(B)** La Paz, Bautista Saavedra, 15°15’0”S, 69°2’50”W, 4549 m, open high Andean area with limestone rocks. **(C)** La Paz, Franz Tamayo, 14°46’39”S, 69°0’35”W, 2550 m, Yungas montane cloud forest. **(D)** Tarija, Aniceto Arce, 21°41’36”S, 64°29’33”W, 2195 m, Tucumano-Boliviano montane forest. **(E)** Santa Cruz, Cordillera, 18°27’29”S, 61°23’1”W, 292 m, Chaqueño forest. **(F)** Beni, Yacuma, 14°51’7”S, 66°20’23”W, 175 m, Los Llanos de Moxos savanna. All photographs by AF.

Specimens of Bolivian lichens suspected of belonging to Lecanoraceae based on their morphology were collected from 2004 to 2019. Collection sites spanned over 4,700 m of elevation and included diverse natural communities across multiple biomes (Navarro and Maldonado, 2002; Navarro and Ferreira, 2007). Habitats ranged from patches of trees in Los Llanos de Moxos savanna below 200 m, to Yungas and Tucumano-Boliviano montane forests at mid-elevation sites, to dry, rocky grasslands of the high Andean Puna at about 4,000 m and subnival vegetation above 4,600 m (Figure 1). Voucher specimens are stored in KRAM and LPB.

### Morphological Studies

Morphology and anatomy were studied by standard techniques. Cross sections of apothecia were mounted in water for observation of apothecium anatomy or in ca. 25% KOH for observation of ascospores. Crystals were observed under polarized light in a compound fluorescence microscope (Nikon Eclipse 80i) and their solubility was tested with KOH and 65% nitric acid. Lichen secondary metabolites were analyzed by thin-layer chromatography (TLC) in solvents A, B’, and C following Culberson and Kristinsson (1970) and Orange et al. (2001). For saxicolous samples, rocks were tested for carbonate minerals with 10% HCl.

### DNA Extraction, PCR, and Sequencing

From a collection of approximately 550 specimens, 235 specimens representing diverse morphotypes and habitats were selected for molecular study. Note that the genus *Lecidella*, although part of Lecanoraceae, was not targeted in this sampling focused on lecanoroid species. Material collected in the years 2015–2018 was kept frozen at −20°C prior to DNA extraction, while collections from earlier years were stored under standard herbarium conditions. Specimens collected in 2019 were extracted when fresh. Apothecia excised from a single thallus—or soralia, if apothecia were absent—were cleaned in sterile, distilled water on a microscope slide. Visible contaminants (e.g., lichenicolous fungi) were removed with ultrathin tweezers and a razor blade. DNA was isolated using either the QIAamp DNA Investigator Kit or DNeasyTM Plant Mini Kit (Qiagen, Germany), depending on the amount of lichen tissue available, following the manufacturer’s instructions.

To conduct a multi-locus phylogenetic analysis of Lecanoraceae, we amplified the mycobiont nuclear ribosomal large subunit (nrLSU), internal transcribed spacer (ITS), mitochondrial small subunit (mtSSU), RNA polymerase II largest (*RPB1*) and second-largest (*RPB2*) subunits, and minichromosome maintenance factor 7 (*MCM7*). The ribosomal RNA (rRNA) loci ITS, mtSSU, and to some extent nrLSU have been frequently sequenced for phylogenetic studies of Lecanoraceae and represent most of the reference data for this family on GenBank. The ITS region, including the ITS1 spacer, 5.8S rRNA, and ITS2 spacer, was amplified with primers ITS1F (Gardes and Bruns, 1993) and either LR3 or ITS4 (Vilgalys and Hester, 1990; White et al., 1990); nrLSU was amplified with primers AL2R and LR6 (Vilgalys and Hester, 1990; Döring et al., 2000); and mtSSU was amplified with primers MSU1 and MSU7 (Zhou and Stanosz, 2001). Thermal cycler conditions for the three rRNA loci were 95°C for 3 min, followed by 35 cycles of 95°C for 40 s, 52°C for 40 s, and 72°C for 150 s, with a final extension at 72°C for 10 min.

Due to the very small amount of DNA present in our extractions, the protein-coding loci *RPB1*, *RPB2*, and *MCM7* were amplified with a nested PCR approach. For *RPB1*, the initial amplification was performed with primers RPB1-Af and either RPB1-Cr or RPB1-Dr (Stiller and Hall, 1997; Matheny et al., 2002). In the first round of PCR, the thermal cycler conditions were 94°C for 10 min, followed by 25 cycles of 94°C for 45 s, 50°C for 50 s, and 72°C for 100 s, with a final extension at 72°C for 10 min. The second round of PCR used forward and reverse primers lecRPB1-F and lecRPB1-R (Table 1), which we designed based on the Lecanoraceae *RPB1* alignment from Zhao et al. (2016). Suitable priming sites were identified by visual inspection of alignments in Mesquite (Maddison and Maddison, 2018) and potential primer sequences were evaluated using online tools^1^,^2^. The thermal cycler conditions for the second round were 94°C for 5 min, followed by 35 cycles of 94°C for 45 s, 55°C for 60 s, and 72°C for 90 s, with a final extension at 72°C for 10 min. For *RPB2*, the first round of PCR used primers fRPB2-5F and fRPB2-7cR (Liu et al., 1999), and thermal cycler conditions were identical to the first-round program for *RPB1*. The second round used primers lecRPB2-6F and fRPB2-7cR. The newly designed forward primer lecRPB2-6F (Table 1) was modified from bRPB2-6F (Matheny, 2005) using Lecanoraceae sequences from Miadlikowska et al. (2014). Thermal cycler conditions for this round were the same as for the first round, except that the number of cycles was increased to 35 and the extension time was reduced to 85 sec per cycle. For *MCM7*, the first round of PCR used primers MCM7-709f and MCM7-1443r, while the second round used MCM7-709f and MCM7-1348r (Schmitt et al., 2009). The thermal cycler programs for both rounds followed Schmitt et al. (2009), except that the number of cycles in the first round was reduced to 25.

**TABLE 1:** Lecanoraceae-specific *RPB1* and *RPB2* primers designed in the present study.

Locus	Primer name	Use	Nucleotide sequence (5’–3’)
*RPB1*	lecRPB1-F	PCR	GAR ACN GTY TGY CAY AAY TGY GGC AAG
	lecRPB1-R	PCR	C RAA YTC RTT NAC NAC RTG NGC NGG
*RPB2*	lecRPB2-6F	PCR	TGG GGN YTR GTM TGY CCD GC
	lecRPB2-seq7R	sequencing	G RTT RTG RTC NGG RAA NGG

Sanger sequencing of PCR amplicons has been shown to reliably recover the dominant photobiont in *Trebouxia*-associated lichens even when other photobiont genotypes are present at low levels (Paul et al., 2018). Photobiont ITS was amplified with primers ITS1T and ITS4T (Kroken and Taylor, 2000) using the thermal cycler program described above for the mycobiont rRNA loci. For a subset of specimens, we also amplified a portion of the Rubisco large subunit (*rbc*L) with primers a-ch-rbcL-203-5’-MPN and a-ch-rbcL-991-3’-MPN (Nelsen et al., 2011). Thermal cycler conditions followed Nelsen et al. (2011). Selection of specimens for *rbc*L sequencing was based on the photobiont ITS results and focused on specimens that were: (1) outliers in the elevation distribution or host breadth of a photobiont clade, (2) photobionts that appeared to be new species-level lineages, (3) species-level photobiont lineages only found in a single specimen, or (4) previously recognized *Trebouxia* lineages for which *rbc*L data were lacking.

PCR amplicons were checked on 1% agarose gels to confirm the presence of a single fragment size and cleaned following an enzymatic cleanup protocol with exonuclease I and shrimp alkaline phosphatase (ThermoFisher Scientific, Waltham, MA, United States). Sanger sequencing was performed by Eurofins Genomics (Louisville, KY, United States). Sequencing reactions used PCR primers except in two cases. Primers mrSSU1 and mrSSU3R (Zoller et al., 1999) were used in place of MSU1 and MSU7 to sequence the mtSSU amplicons. A newly designed primer, lecRPB2-seq7R (Table 1) was used in place of fRPB2-7cR for sequencing the *RPB2* amplicons. Forward and reverse sequence reads were assembled, trimmed, and checked for base-calling errors in SeqMan Pro (DNASTAR, Madison, WI, United States) or Geneious Prime 2021.0.3^3^. When ITS and partial nrLSU were sequenced as one amplicon, they were split using the program ITSx (Bengtsson-Palme et al., 2013). Sequences were checked for contamination or specimen misidentification using two complementary methods: (1) BLAST with the NCBI nucleotide database and (2) placement in a phylogeny of Lecanoromycetes with T-BAS (Miadlikowska et al., 2014; Carbone et al., 2017, 2019). GenBank numbers for all newly obtained sequences are given in Supplementary Data Sheet 1.

### Alignments and Phylogenetic Analyses

We compiled a reference dataset (Supplementary Data Sheet 2) of Lecanoraceae and outgroups based on taxa included in Miadlikowska et al. (2014) and Zhao et al. (2016). In general, reference taxa were only included if multiple loci, including protein-coding loci, were available. The families Parmeliaceae (represented by one species each of *Protoparmelia* and *Letharia*) and Gypsoplacaceae (represented by two species of *Gypsoplaca*) were used as outgroups. Our Lecanoraceae dataset included several species groups within *Lecanora*, *Palicella*, *Lecidella*, *Protoparmeliopsis*, *Rhizoplaca*, *Myriolecis*, the “*Lecanora*” *polytropa* group, and the “*Lecanora*” *saligna* group, as well as *Haematomma*, *Ramboldia*, and *Miriquidica*. Reference sequences were checked for specimen misidentifications or other metadata errors using BLAST with the NCBI nucleotide database.

The mycobiont rRNA loci (mycobiont ITS, nrLSU, and mtSSU) were initially aligned using the MAFFT online server^4^ with the G-INS-1 option (Katoh et al., 2019). The alignments were corrected by eye in Mesquite (Maddison and Maddison, 2018) and introns and ambiguously aligned regions were delimited manually and excluded from downstream analyses (Lutzoni et al., 2000). Protein-coding loci were aligned by translated amino acid in Mesquite. Introns were delimited manually and excluded from downstream analyses.

Each single-locus alignment was used as input for a maximum likelihood phylogenetic analysis in IQ-TREE version 2.1.2 (Nguyen et al., 2015; Chernomor et al., 2016) run on the CIPRES server (Miller et al., 2010). We performed 5,000 ultrafast bootstrap pseudoreplicates to calculate support for each single-locus tree (Hoang et al., 2018). We inspected the resulting trees for well-supported conflicts (≥95% ultrafast bootstrap; Hoang et al., 2018) among the six mycobiont loci, which were then concatenated to form a single dataset. The concatenated mycobiont alignment was used as input for a partitioned maximum likelihood analysis in IQ-TREE. We ran IQ-TREE with the -p and -m MFP + MERGE options so that ModelFinder (Kalyaanamoorthy et al., 2017) would both optimize the partitioning scheme and find the best-fitting substitution model for each partition. The input partitioning scheme divided the concatenated alignment by locus and by codon position for the protein-coding loci. The final partitioning schemes and substitution models used in this analysis are provided in Table 2. We performed 5,000 ultrafast bootstrap pseudoreplicates to calculate bipartition support for the tree topology.

**TABLE 2:** Statistics on the alignments for Lecanoraceae (mycobiont) after the removal of introns and ambiguously aligned regions.

Locus	Length	Invariable (%)	PI (%)	Missing taxa (%)	Model/Partition
ITS	252	144(57)	69(27)	14(9)	1
nrLSU	1024	701(68)	214(21)	35(23)	1
mtSSU	789	531(67)	172(22)	29(19)	2
*RPB1*	630	298(47)	297(47)	42(27)	3:3:4
*RPB2*	744	329(44)	367(49)	56(36)	3:2:4
*MCM7*	537	269(50)	249(46)	102(66)	3:2:4

*The full concatenated alignment included 154 taxa and 3,976 sites. PI, parsimony-informative sites. Substitution models were as follows: 1, TN + F + R4; 2, HKY + F + R3; 3, GTR + F + R3; 4, TIM2e + I + G4. Models for protein-coding loci are given as first:second:third codon positions. Loci and codon positions with the same substitution model were fused into a single partition.*

We performed two phylogenetic analyses with the photobiont data. First, all sequences were included in a global ITS-*rbc*L alignment for *Trebouxia* to assign each sample to one of the major *Trebouxia* clades. We used the ITS and *rbc*L sequences curated by Muggia et al. (2020) as a reference dataset, with slight modifications. These sequences are listed in the file “Supplementary Data 2” from Muggia et al. (2020). We removed sequence L138 from the *Trebouxia* clade S ITS alignment and sequences L1107, L1360, L1401, L1417, L1425, L1504, AJ007387, AJ969549, AJ249482, and 9493 from the *Trebouxia* clade A ITS alignment because they were missing a significant portion of ITS1 or ITS2. None of these ITS sequences had a corresponding *rbc*L sequence. L1107 and 9493 were the only representatives of the putative species A37 and A49, respectively, but preliminary analyses indicated that our new sequences were not closely related to these taxa. We also added all ITS and *rbc*L reference sequences for *Trebouxia* clade D from “Supplementary Table 5” of Xu et al. (2020). Representatives from three other genera of Trebouxiophyceae were used as outgroup taxa: *Asterochloris*, GenBank accession JN573844; *Myrmecia*, KM462861; and *Vulcanochloris*, KR952313. Only *rbc*L was included for the outgroup taxa because ITS was not alignable across the four genera.

ITS sequences were initially aligned with MAFFT using the G-INS-1 option and regions that were not conserved across *Trebouxia* were excluded by eye in Mesquite; *rbc*L sequences were aligned by translated amino acid in Mesquite. Phylogenies for each locus were inferred as described above for the mycobiont loci. The two alignments were concatenated after inspecting single-locus trees for well-supported conflicts. We used the concatenated dataset as input for a maximum likelihood phylogenetic analysis in IQ-TREE. Model selection, tree inference, and bootstrapping parameters were the same as described above for the concatenated mycobiont dataset, with the initial partitioning scheme dividing the alignment by locus and further splitting *rbc*L by codon position.

For the second set of analyses, we performed a separate phylogenetic analysis for each major clade of *Trebouxia* represented in our sampling. The genus-wide alignment allowed unknown sequences to be assigned to one of the major clades, but because variable regions of ITS1 and ITS2 had to be excluded, relationships within these clades were often poorly resolved. For each major clade, reference taxa and sample sequences were extracted from the global ITS and *rbc*L alignments. The ITS alignments were corrected by eye in Mesquite and ambiguous regions were re-delimited. One *rbc*L sequence from each of the other major clades of *Trebouxia* was retained for the outgroup. After checking for well-supported conflicts between the two loci as described above, we concatenated the ITS and *rbc*L alignments and performed a maximum-likelihood analysis in IQ-TREE. Model selection, tree inference, and bootstrapping parameters were the same as described above for the concatenated mycobiont dataset. The initial partitioning scheme divided the alignment by locus and further split the ITS by region (ITS1, 5.8S, and ITS2) and *rbc*L by codon position. The final partitioning schemes and substitution models used for these analyses are provided in Table 3.

**TABLE 3:** Statistics on the alignments for *Trebouxia* (photobiont) after the removal of introns and ambiguously aligned regions.

Clade	ITS			*rbc*L	
	Length	PI (%)	Model/Partition	PI (%)	Model/Partition
A	516	150(29)	1:2:1	83(11)	2:2:3
C	514	129(25)	4:5:4	121(15)	5:5:6
I	505	101(20)	7:5:7	86(11)	5:5:8
S	552	123(22)	9:5:9	39(5)	5:5:3

*PI, parsimony-informative sites. The rbcL alignment was 789 bases long for all clades and had no excluded positions. Substitution models were as follows: 1 = TVMe + R3; 2 = K2P + R2; 3 = TIM3 + F + R3; 4 = SYM + R3; 5 = K2P + I; 6 = TIM2 + F + R3; 7 = TPM2 + F + G4; 8 = TIM2 + F + I; 9 = TIM3e + G4. Models are given as ITS1:5.8S:ITS2 for the ITS and first:second:third codon positions for rbcL. ITS regions and codon positions with the same substitution model were fused into a single partition.*

To explore intraspecific diversity in a subset of putative *Trebouxia* species, we inferred haplotype networks in R version 3.6.1 (R Core Team, 2019) using the packages ape (Paradis and Schliep, 2019) and pegas (Paradis, 2010). Sites with ambiguous bases were excluded from the haplotype analysis.

### Classification of Mycobionts and Photobionts

Our preliminary delimitation of mycobiont species was based on morphology, chemistry, and the inferred phylogeny. We used the program bPTP (Zhang et al., 2013) to inform our species delimitation, using the concatenated maximum likelihood phylogeny as input and running the Markov chain Monte Carlo (MCMC) analysis for 500,000 generations, sampling every 500 generations^5^. The first 25% of samples were discarded as burn-in.

Based on the ITS-*rbc*L phylogeny, photobiont sequences were assigned to a clade and putative species based on the framework from Muggia et al. (2020). Specimens that were phylogenetically distinct from any previously delimited species were considered to represent novel putative species.

### Ecological Analyses

To better understand the ecological diversity across the elevation gradient, we extracted 19 bioclimatic variables from the WorldClim database (Fick and Hijmans, 2017) for each sampling locality. Bioclimatic variables were sampled at a resolution of 10 arc-minutes using the R packages sp (Pebesma and Bivand, 2005) and raster (Hijmans, 2021). We used the hclust function to perform a hierarchical clustering of the 19 bioclimatic variables, which was visualized as a dendrogram. Vegetation types for each site were classified with field locality descriptions and vegetation maps from Navarro and Ferreira (2007).

We analyzed patterns of species richness (α diversity) and turnover (β diversity) along the elevation gradient. From the original data for each lichen specimen (Supplementary Data Sheet 1), we prepared inferred presence/absence matrices for the mycobiont and photobiont species. The elevation gradient was split into 500 m bins starting at sea level and extending to 4,999 m. For each Lecanoraceae or *Trebouxia* species, we noted the lowest and highest elevations at which the species was found, and coded the species as “present” for all bins included in that range. For example, a *Trebouxia* species found at 2,400, 3,050, and 3,600 m would be coded as present from 2,000 to 3,999 m even though it was absent from the 2,500 to 2,999 m bin. The Sørensen index of dissimilarity was calculated for all pairwise comparisons between elevation bins using the vegdist function in the R package vegan (Oksanen et al., 2020).

## Results

### Sequenced Specimens

We obtained DNA sequence data from approximately half of the 235 specimens for which extractions were performed. Most of the specimens that failed to yield any sequence data were those collected before 2015 and stored under standard herbarium conditions. Of the specimens from which we obtained sequence data, approximately ten were not Lecanoraceae and were excluded from further analysis. A further ca. 20 specimens yielded only mycobiont or only photobiont sequences; the sequences from specimens without paired mycobiont–photobiont data were not used for the present paper. Finally, five specimens were excluded from the analysis because of well-supported conflicts between loci or discordance between molecular and morphological data. A total of 89 specimens with both mycobiont and photobiont data were used for the remaining analyses. We generated over 500 new DNA sequences from this group of specimens: 83 mycobiont ITS, 68 nrLSU, 79 mtSSU, 70 *RPB1*, 64 *RPB2*, 22 *MCM7*, 89 photobiont ITS, and 27 *rbc*L.

### Alignments

Summary statistics for the Lecanoraceae and *Trebouxia* alignments are presented in Tables 2, 3, respectively. For the mycobiont, almost all of ITS1 and a large fraction of ITS2 could not be aligned across Lecanoraceae and were excluded from the phylogenetic analysis. The three protein-coding loci contained the greatest number of parsimony-informative sites in absolute terms and relative to their length (Table 2). For the *Trebouxia* sequences, most of ITS1 and ITS2 could be aligned within each major clade. ITS provided most of the parsimony-informative sites for all four *Trebouxia* single-clade alignments, while *rbc*L varied from being nearly as informative as ITS in *Trebouxia* clade C to being minimally informative in *Trebouxia* clade S (Table 3). Complete alignments with excluded regions delimited are available in the supplementary materials (Supplementary Data Sheets 3–8).

### Lecanoraceae and *Trebouxia* Phylogeny and Biodiversity

**FIGURE 2:**
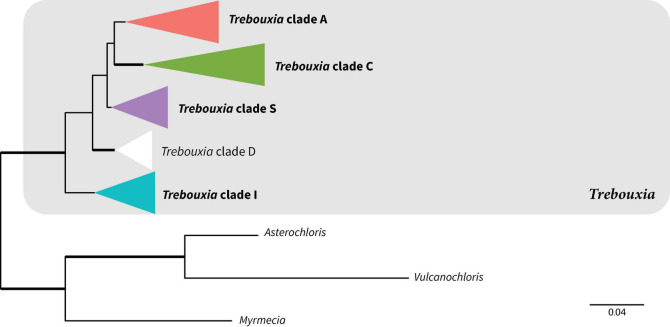
Summary phylogeny showing relationships among major clades of *Trebouxia* based on maximum likelihood analysis of the ITS-*rbc*L dataset. Bold clade names indicate clades represented in our Bolivian sampling. Bold branches indicate UFboot2 support ≥ 95. Scale represents substitutions per site. The color scheme introduced here will be used for these *Trebouxia* clades throughout the rest of the paper. For the full tree with all tip names and bootstrap values, see Supplementary Figure 1.

Photobionts from members of the Lecanoraceae collected in Bolivia represented approximately 21 putative species in four of the five major clades within *Trebouxia* (Figure 2 and Supplementary Figure 1). The majority of photobionts were phylogenetically nested within previously delimited species, but six lineages appear to represent new species-level taxa in *Trebouxia* clades A, C, and I (Supplementary Figures 2–4). All samples from *Trebouxia* clade S corresponded to previously delimited species (Supplementary Figure 5). No samples were from *Trebouxia* group D (Supplementary Figure 1).

**FIGURE 3:**
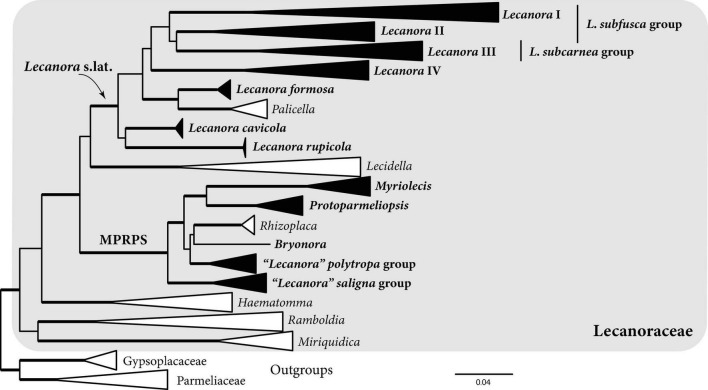
Summary phylogeny showing relationships within Lecanoraceae based on maximum likelihood analysis of the six-locus dataset. The clade names *Lecanora* I–IV, *Lecanora* s.lat., and MPRPS (*Myriolecis*, *Protoparmeliopsis*, *Rhizoplaca*, “*L*.” *polytropa*, “*L*.” *saligna*) will be used throughout the paper in the sense shown here. Bold clade names and filled triangles indicate clades represented in our Bolivian sampling. Bold branches indicate UFboot2 support ≥ 95. Scale represents substitutions per site. For the full tree with all tip names and bootstrap values, see Supplementary Figure 12.

The single-locus trees for the mycobiont ITS, nrLSU, and mtSSU had some well-supported nodes near the tips, but deeper relationships were for the most part poorly supported (Supplementary Figures 6–8). *RPB1*, *RPB2*, and *MCM7* were better able to resolve deeper relationships in Lecanoraceae, with *RPB1* yielding the best-supported tree of any single locus (Supplementary Figures 9–11). The maximum likelihood tree from the concatenated six-locus dataset (Figure 3 and Supplementary Figure 12) included strong support for many putative genus- and subgenus-level clades and some relationships among those clades, but the backbone was poorly supported in several regions of the tree. For example, the relationships among *Haematomma*, *Miriquidica*, *Ramboldia*, and the rest of the family were not well supported, nor was the position of *Lecidella*. However, none of our mycobiont specimens belonged to these genera.

**FIGURE 4:**
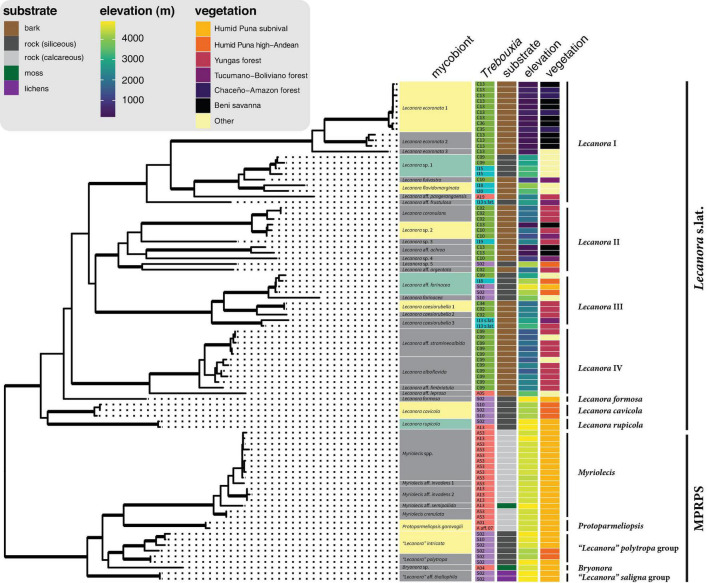
Photobiont association, substrate, elevation, and vegetation type in the context of the mycobiont phylogeny. The tree is from the same six-locus analysis as Figure 3 and Supplementary Figure 12; reference taxa were removed in R with the drop.tip function in ape and data were added to the tree with the package ggtree (Yu et al., 2017). Bold branches indicate clades with UFboot2 support ≥ 95 in Supplementary Figure 12. Lecanoraceae species highlighted in green were found with photobionts from more than one major clade of *Trebouxia*. Lecanoraceae species highlighted in yellow were found with multiple *Trebouxia* species from the same clade. Colors used for *Trebouxia* clades are the same as in Figure 2. Species-level relationships within *Myriolecis* are not well-resolved in this analysis.

**FIGURE 5:**
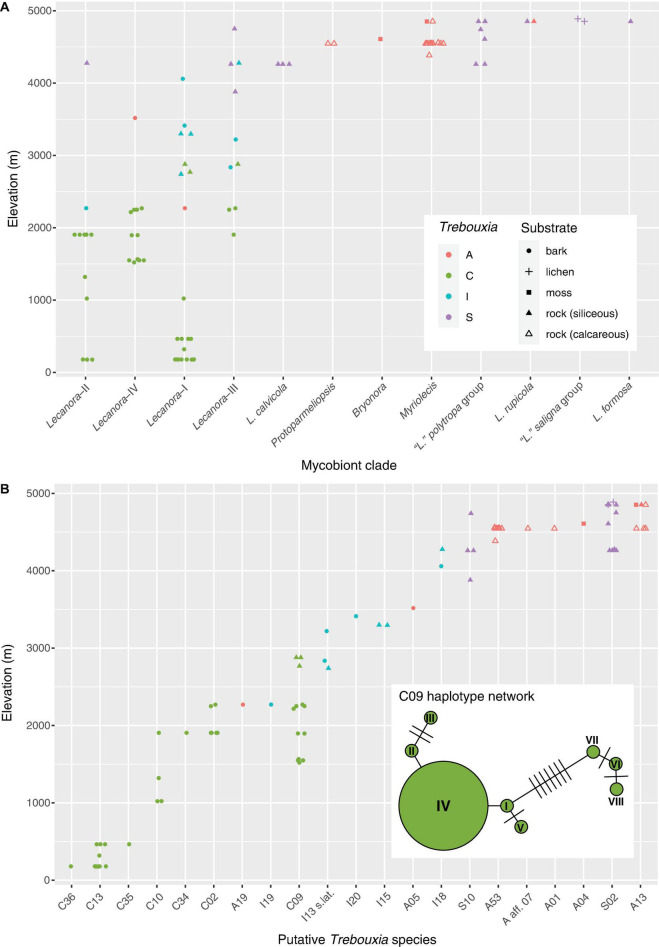
Elevation distribution of Lecanoraceae mycobionts and their *Trebouxia* photobionts. **(A)** Elevation distribution of the major mycobiont clades, showing *Trebouxia* clades and substrates. Note that photobiont turnover across the elevation gradient is apparent for Lecanoraceae as a whole and within individual mycobiont clades. There are relatively few samples from the 3,000 to 4,000 m range because specimens for molecular study were originally selected for a taxonomic study of Lecanoraceae. Although we have many collections from this altitudinal range, they were not particularly diverse morphologically and therefore were not sampled heavily in our sequencing. **(B)** Elevation distribution of putative *Trebouxia* species. A single photobiont species typically occupies 500–1,000 m of the elevation gradient. Inset shows haplotype network for *Trebouxia* C09, which occupies 1,500 m of the gradient. The haplotypes clustered on the left occur across the elevation range of this species, while those clustered on the right only occur at the bottom of the range.

Sampled mycobionts belong to two well-supported clades within Lecanoraceae (Figure 3). The first clade, which we call *Lecanora* s.lat., includes the *Lecanora subfusca* group (*Lecanora* clades I and II), *L. subcarnea* group (*Lecanora* clade III), a group we call *Lecanora* clade IV, *L. formosa* and *Palicella*, *L. cavicola*, and *L. rupicola*. None of the relationships among these clades were well supported. Species of *Lecanora* s.lat. were found predominantly on bark at low- to mid-elevation sites, but some collections were from rock at high elevation (Figure 4). The high-elevation, saxicolous specimens mostly belonged to *L. rupicola*, *L. cavicola*, and *L. formosa*, but also included taxa from *Lecanora* clades II and III (Figures 4, 5A).

The second major clade, which we call the MPRPS clade for lack of a formal name, included high-elevation collections from *Myriolecis*, *Protoparmeliopsis*, the “*Lecanora*” *polytropa* group, *Bryonora*, and the “*Lecanora*” *saligna* group. MPRPS also includes *Rhizoplaca*, although none of our collections belong to that genus (Figure 3). *Myriolecis* and *Protoparmeliopsis* form a well-supported clade, and the “*L.*” *saligna* group is a well-supported sister to the rest of the MPRPS clade, but the phylogenetic placements of the “*L.*” *polytropa* group, *Rhizoplaca*, and *Bryonora* are uncertain. Except for the single specimen of *Bryonora* and one *Myriolecis* specimen that were found on moss and two specimens of the “*L.*” *saligna* group that were lichenicolous lichens, species in the MPRPS clade occurred on rock (Figure 4).

Based on morphology, chemistry, and the molecular phylogeny, our sampling included approximately 27 species in *Lecanora* s.lat. and approximately 10 species in the MPRPS clade (Figure 4). The tree topology and species delimitation analysis suggest that several of the morphospecies contain multiple cryptic species. For example, there are three well-supported lineages on long branches in the morphospecies *Lecanora ecoronata* (*Lecanora* clade I), and the specimens identified as *L. caesiorubella* were distributed across three lineages in *Lecanora* clade III that did not form a monophyletic group (Figure 4).

### Photobiont Ecology and Mycobiont Associations

The distribution of *Trebouxia* species was structured by elevation, substrate, and mycobiont identity (Figures 4, 5). Species of the MPRPS clade were always found with photobionts from *Trebouxia* clades A and S. *Myriolecis*, *Protoparmeliopsis*, and the single specimen of *Bryonora* were exclusively associated with *Trebouxia* clade A, while the “*Lecanora*” *polytropa* and “*L.*” *saligna* groups occurred only with *Trebouxia* clade S. All of these mycobiont genera were restricted to high elevations (Figure 5A). This pattern is also related to substrate chemistry: Saxicolous species of *Myriolecis* and *Protoparmeliopsis* grew on calcareous rock, while species of the “*L*.” *polytropa* group and the lichen hosts of members of the “*L*.” *saligna* group occurred on siliceous rock (Figure 4). All of these mycobiont genera were restricted to high-elevation sites (Figure 5A).

Species of the *Lecanora* s.lat. clade were found in association with *Trebouxia* clades A, C, I, and S. There was a pronounced turnover in photobiont clade along the elevation gradient, with *Trebouxia* clade C at low to mid-elevations (up to ca. 3,000 m), clade I mostly at upper mid elevations (ca. 2,000–3,500 m), and clade S at high elevations (above 3,500 m) (Figure 5A). *Trebouxia* clade A was only rarely associated with *Lecanora* s.lat., always above 2,000 m (Figure 5A).

Clades within *Lecanora* s.lat. occupied varying portions of the total elevation gradient (Figure 5A). *Lecanora* clade I was only absent from the highest elevations, while *Lecanora* clade III was missing at low elevations; *Lecanora* clade IV occupied intermediate elevations. *Lecanora* clade II was primarily at low elevations, except for a single high elevation species. *Lecanora* clades I–IV were each associated with photobionts from at least two, and often three, *Trebouxia* clades. The same elevational turnover of photobiont clades that is apparent for Lecanoraceae as a whole also occurs within these smaller clades; see, for example, *Lecanora* clades I and III (Figure 5A).

At the level of putative *Trebouxia* species, there is considerable variation in the level of specificity exhibited toward habitat and mycobiont (Figures 4, 5B). Most *Trebouxia* species occupied a 500–1,000 m subset of the elevation gradient (Figure 5B). Some taxa occurred only with a single mycobiont clade in that narrow elevation range. For example, *Trebouxia* C13 was only found below 500 m with species of *Lecanora* clade I, while *Trebouxia* A53 only occurred with species of *Myriolecis* at about 4,500 m (Figure 4). Other photobionts were generalists with respect to mycobiont but were only found in a specific habitat. *Trebouxia* A13, S02, and S10 occurred with mycobiont species from both the *Lecanora* s.lat. and MPRPS clades, but nearly always above 4,000 m (Figure 4). On the other hand, some photobionts exhibited substantial niche breadth for both mycobiont host and elevation. *Trebouxia* C09, the most broadly distributed photobiont species in our sampling, was associated with species from *Lecanora* clades I–IV and occurred on both bark and rock from 1,500 to 3,000 m (Figures 4, 5B).

**FIGURE 6:**
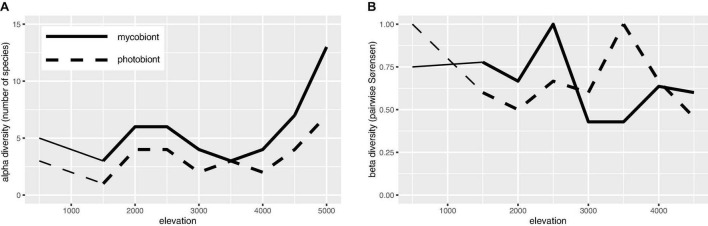
Species richness and β-diversity of Lecanoraceae and *Trebouxia* along the elevation gradient. **(A)** Number of Lecanoraceae (solid line) and *Trebouxia* (dashed line) species observed and inferred for 500 m elevation bins. **(B)** Pairwise comparisons of 500 m elevation bins by the Sørensen index of dissimilarity, where 0 means all species are shared and 1 means no species are shared. Note that each partner has a mid- to mid-high-elevation threshold not spanned by any species, but this threshold is not the same for mycobiont and photobiont. Comparisons below 1,500 m (reduced line weight) may be unreliable because of the absence of samples from the 500–999 m elevation band.

Numbers of mycobiont and photobiont species were tightly linked across the elevation gradient, with the number of mycobiont species nearly always greater; both Lecanoraceae and *Trebouxia* had the greatest number of species at high elevation (Figure 6A). Species turnover was consistently high across the elevation gradient for both partners (Figure 6B). Each partner had a mid- to mid-high-elevation threshold across which no species were shared, but this threshold differed between Lecanoraceae (2,500 m) and *Trebouxia* (3,500 m) (Figure 6B).

### Photobiont Switching

Three mycobiont species were associated with photobionts from two or more *Trebouxia* clades (Figure 4). *Lecanora* sp. 1 (*n* = 4) occurred on sandstone in open areas between 2,700 and 3,300 m. Below 3,000 m, this species was found with *Trebouxia* C09, while above 3,000 m it was found with *Trebouxia* I15. *Lecanora rupicola* (*n* = 2) was found in association with *Trebouxia* A13 and S02 at a single high-elevation site, where it grew on siliceous rocks. *Lecanora* aff. *farinacea* (*n* = 4) occurred with *Trebouxia* C09 at ca. 2,900 m and with *Trebouxia* I18 and S02 at ca. 4,250 m. All four collections were from siliceous rocks, either sandstone at the lower site or schist at the upper sites.

A larger number of mycobiont species associated with multiple photobionts within one of the major *Trebouxia* clades (Figure 4). “*Lecanora*” *intricata* (*n* = 4) and *L. cavicola* (*n* = 3) each associated with *Trebouxia* S02 and S10. At one site at ca. 4,260 m, *L. cavicola* was found with both photobionts. All specimens of these two species were collected from siliceous rocks in open vegetation above 4,000 m. *Protoparmeliopsis garovaglii* (*n* = 2), which was only collected at a single site at ca. 4,550 m, on limestone, occurred with *Trebouxia* A01 and A aff. 07. *Lecanora caesiorubella* 1 (*n* = 2) occurred with *Trebouxia* C34 at ca. 1,900 m and *Trebouxia* C02 at 2,250 m; both sites were in Yungas forest. *Lecanora ecoronata* 1 (*n* = 9) was predominantly associated with *Trebouxia* C13, but at the same savanna sites also occurred with *Trebouxia* C35 and C36. *Lecanora* sp. 2 (*n* = 3) occurred with *Trebouxia* C13 at the bottom of its range and C10 at the top of its range. *Lecanora flavidomarginata* (*n* = 2) occurred with *Trebouxia* I18 and I20, at ca. 4,000 m and ca. 3,400 m, respectively.

Haplotypes of *Trebouxia* C09 fell into two clusters (Figure 5B). One cluster spanned the entire elevation range of this putative species, while the other occurred only in the lower portion of its range. *Lecanora* aff. *stramineoalbida* was associated with both haplotype clusters.

## Discussion

### Mycobiont Phylogeny

Phylogenetic studies of Lecanoraceae have long been bedeviled by a poorly supported backbone (Grube et al., 2004; Pérez-Ortega et al., 2010; Zhao et al., 2016; Yakovchenko et al., 2019; Davydov et al., 2021). Most of these studies, including recent publications, have used ITS and mtSSU to infer the phylogeny of Lecanoraceae, but those loci are most useful for resolving relationships within genera or species groups and cannot resolve deeper relationships across the entire family. Zhao et al. (2016) obtained better support with a six-locus dataset, but their analysis excluded many morphologically distinct species groups for which the protein-coding loci were lacking. In the present study, we used the same six loci as Zhao et al. (2016): ITS, nrLSU, mtSSU, *RPB1*, *RPB2*, and *MCM7*. We expanded their taxon sampling, bringing in additional clades that were missing from their six-locus analysis: *Bryonora*, *Lecanora* IV, *Lecanora cavicola*, *Lecanora rupicola*, and the “*Lecanora” saligna* group. Despite this expanded sampling, we found that several regions of the phylogeny remain poorly supported.

One particularly difficult problem in Lecanoraceae systematics is the delimitation of *Lecanora* s.str. In the present paper, we have sidestepped this issue by referring to a well-supported *Lecanora* s.lat. and well-supported clades nested within it, without making a taxonomic judgment about what should be included in *Lecanora* s.str. If morphologically distinctive groups such as *Palicella*, *Pulvinora*, and the *Lecanora rupicola* group are to be excluded from *Lecanora* s.str., as various authors have proposed (Grube et al., 2004; Rodriguez Flakus and Printzen, 2014; Davydov et al., 2021), *Lecanora* s.str. should ideally be tied to a well-supported node within our *Lecanora* s.lat. Our analysis recovered no such node deeper than the species groups delimited in Figure 3. For example, the *Lecanora subfusca* group as traditionally circumscribed (*Lecanora* clades I and II; Zhao et al., 2016) was not well supported in our analysis (Figure 3 and Supplementary Figure 12). Further research, potentially with genomic data, will be required to stabilize the genus-level nomenclature of Lecanoraceae.

Based on our results and the existing literature, we recommend sequencing at a minimum ITS, mtSSU, and *RPB1* in order to place a specimen within the phylogeny of Lecanoraceae. The new primers and nested PCR protocols we describe in this paper can be used to sequence *RPB1* and *RPB2* even from extractions with a very small quantity of DNA. In this study, we have substantially increased the number of *RPB1*, *RPB2*, and *MCM7* sequences available for Lecanoraceae, which should facilitate the use of these loci in future phylogenetic studies of this family. The inclusion of *Bryonora* in our multilocus dataset illustrates how the protein-coding loci can clarify relationships in Lecanoraceae. *Bryonora* was previously represented on GenBank by a single ITS sequence from Grube et al. (2004), and its phylogenetic position was unknown. The phylogenetic analysis of Grube et al. (2004) placed it outside Lecanoraceae, albeit without support. Our results, which included ITS, nrLSU, mtSSU, *RPB2*, and *MCM7* sequences for a specimen of *Bryonora*, strongly support *Bryonora* as a distinct genus belonging to the MPRPS clade.

Although there have been morphological studies on Lecanoraceae in Bolivia (Śliwa et al., 2013, 2014; to a minor extent, Guderley, 1999), ours is the first study to generate molecular data for Bolivian Lecanoraceae and one of the only molecular phylogenetic studies of tropical Lecanoraceae (Kirika et al., 2012; Papong et al., 2013). These data will be used in forthcoming publications on the systematics of Lecanoraceae. As a resource for the community, we have made our Lecanoraceae and *Trebouxia* trees and alignments available on the T-BAS online portal^6^ to facilitate the phylogenetic placement of specimens from these clades (Carbone et al., 2017, 2019).

### *Trebouxia* Diversity and Ecology

We found Lecanoraceae mycobionts from Bolivia in association with four of the five major clades of *Trebouxia*, with only *Trebouxia* clade D absent from our sampling. Clades A, I, and S have been recorded with Lecanoraceae in numerous previous studies (Beck, 1999; Blaha et al., 2006; Guzow-Krzemińska, 2006; Hauck et al., 2007; Pérez-Ortega et al., 2012; Ruprecht et al., 2012, 2020; Leavitt et al., 2015, 2016; Voytsekhovich and Beck, 2016; Singh et al., 2019; Muggia et al., 2020; Wagner et al., 2020). Lecanoraceae species have not previously been reported to associate with *Trebouxia* clade C. Our results show that species of *Lecanora* s.lat.—specifically *Lecanora* clades I–IV—frequently associate with *Trebouxia* clade C photobionts in low to mid-elevation habitats (Figure 5A). That this association has not been seen before is a result of the dearth of molecular phylogenetic studies on tropical *Lecanora*. Our results reinforce the importance of *Trebouxia* clade C photobionts in tropical lichens. The prevalence of *Trebouxia* clade C photobionts in the tropics has been recognized for nearly two decades (Helms, 2003; Cordeiro et al., 2005), but it was not until recently that the species richness of this clade was well understood (Muggia et al., 2020).

Several *Trebouxia* ecological patterns seen in other regions and with other mycobiont taxa are evident in our results. These include the association of *Trebouxia* clade A with calcareous rock (Helms, 2003) and open, intermittently humid Andean vegetation (Vargas Castillo and Beck, 2012), as well as the association of *Trebouxia* clade S with high elevation (Blaha et al., 2006; Muggia et al., 2008). In an analysis of over 6,000 publicly available sequences of lichenized *Trebouxia*, originally sampled from around the world and associated with diverse mycobiont hosts, Nelsen et al. (2021) found that *Trebouxia* clade I photobionts occupy a bioclimatic space intermediate between clade C and clade S, while clade A—the most speciose lineage in *Trebouxia*—overlaps with all three. Our results confirm this pattern (Figures 5A,B) and show that it can be observed on a regional scale within the symbionts of a single mycobiont family.

Along an elevation gradient from ca. 700–2,100 m in Europe, Dal Grande et al. (2018) found that *Trebouxia* photobionts of the mycobiont genus *Lasallia* had larger altitudinal ranges at higher elevations. We saw no evidence for this pattern across the entire elevation range represented in our sampling (Figure 5B). It is possible that greater inter-biome variation in climate and vegetation along our nearly 5,000 m elevation gradient leads to species turnover that overrides any such pattern.

Our photobiont data included putative new species of *Trebouxia* in clades A, C, and I (Supplementary Figures 2–4). In clade S, which is globally less diverse than the other three clades (Muggia et al., 2020), all of our specimens belonged to previously delimited species (Supplementary Figure 5). Most of the putative novel species were recovered from a single specimen (C34, C35, C36, I19, and I20) (Figure 5B). The exception is A53, which we found in 11 specimens of *Myriolecis* across four localities in the humid puna subnival vegetation (Figures 4, 5). A53 was always found in lichen thalli growing on exposed, calcareous rock (Figure 4).

Several *Trebouxia* species we found in our sampling were previously known primarily, or exclusively, from east Africa (see below). Shared *Trebouxia* genotypes between Bolivia and Kenya have been previously reported (Lutsak et al., 2016), highlighting that long-distance dispersal probably plays a major role in the distribution of lichenized algae (Rolshausen et al., 2020).

### Notes on Selected *Trebouxia* Species

A01 — We found this species associated with a single thallus of *Protoparmeliopsis garovaglli*. Guzow-Krzemińska (2006) recorded this photobiont in association with *P. muralis* in Europe, and Leavitt et al. (2015) found it associated with species of *Protoparmeliopsis*, *Lecanora*, *Rhizoplaca*, and *Xanthoparmelia*.

A04 — We recovered *Trebouxia* A04 from a single specimen of *Bryonora*. This is the first photobiont DNA sequence obtained from a lichen thallus of this mycobiont genus.

A05 — This species was found in association with a species of *Lecanora* clade IV from ca. 3,500 m. It has otherwise been reported from various species of Parmeliaceae: *Hypotrachyna* and *Punctelia* in Kenya (Muggia et al., 2020) and *Oropogon* in Central and South America (Leavitt et al., 2015).

A13 — This species is globally distributed (De los Ríos et al., 2002; Blaha et al., 2006; Guzow-Krzemińska, 2006; Muggia et al., 2008, 2014; Nyati et al., 2014; Leavitt et al., 2015; Voytsekhovich and Beck, 2016).

A19 — We provide the first *rbc*L sequence from this previously delimited species (Muggia et al., 2020).

A53 — This is the most common novel putative species we recovered in our sampling (Figure 4). It was always found in association with species of *Myriolecis* growing on calcareous rock.

C02 — We provide the first *rbc*L sequence from this previously delimited species (Muggia et al., 2020).

C09 — This species was previously reported from multiple genera of Parmeliaceae in Kenya and from *Tephromela* in Kenya, Russia, and Peru (Muggia et al., 2014, 2020). In our sampling, *Trebouxia* C09 was found in association with species of *Lecanora* s.lat. It is clearly a generalist with respect to mycobiont host and substrate, occurring with species of *Lecanora* clade IV on bark at the lower end of its altitudinal range, then with species from clades I, II, and III, on bark and on rock, at the upper end of its range (Figures 4, 5B).

C10 — This species was previously reported from multiple genera of Parmeliaceae in Kenya and *Parmotrema tinctorum* in Japan (Ohmura et al., 2006; Muggia et al., 2020). In our sampling, *Trebouxia* C10 was only found in association with species of *Lecanora* clades I and II (i.e., the *Lecanora subfusca* group) (Figure 4).

C13 — This species is known from three specimens of *Parmotrema* and *Canoparmelia* from Kenya (Muggia et al., 2020). In our sampling, *Trebouxia* C13 was only found in association with species of *Lecanora* clades I and II (i.e., the *Lecanora subfusca* group) (Figure 4).

C34 — This novel putative species was also found by Kosecka et al. (2021b). See discussion below.

C35 and C36 — These OTUs form a novel lineage sister to the rest of *Trebouxia* clade C (Supplementary Figure 3). They were both found in association with *Lecanora ecoronata* 1 in the low-elevation savanna. The phylogenetic position of these species is supported by both ITS and *rbc*L, but additional investigations of the ultrastructure of these species should be conducted to evaluate whether they would be better treated as a new major clade (i.e., if they do not have a corticola-type pyrenoid).

I13 s.lat. — We found several specimens associated with *Trebouxia* I13 but, despite having both ITS and *rbc*L data, the clade is not well supported (Supplementary Figure 4). This region of the *Trebouxia* clade I tree, which also includes I17, I18, I19, and I20, appears to be primarily tropical (Muggia et al., 2020) and requires further phylogenetic study.

I15 — We provide the first *rbc*L sequence from this previously delimited species. *Trebouxia* I15 was reported from *Punctelia* and *Hypotrachyna* in Kenya (Leavitt et al., 2015; Muggia et al., 2020).

I18 — This species was reported from *Hypotrachyna* in Peru and Kenya and *Tephromela* in Peru (Muggia et al., 2014, 2020).

I19 and I20 — These novel putative species were also found by Kosecka et al. (2021b). See discussion below.

S02 — This species is a generalist with respect to mycobiont host, associating with species of multiple genera within both the *Lecanora* s.lat. and MPRPS clades.

S10 — This OTU corresponds to the formally described species *Trebouxia simplex* (Tschermak-Woess, 1978), the type of which was originally isolated from *Chaenotheca* in Europe, and has also been reported from *Bryoria* in North America and Europe (Lindgren et al., 2014) and *Lasallia* in Europe (Sadowska-Deś et al., 2014). Our results confirm that S10/*T. simplex* is a generalist with respect to mycobiont host, associating with species from both the *Lecanora* s.lat and MPRPS clades.

### Multiple Photobionts in a Single Thallus

Although intra-thalline genetic diversity has been documented in lichenized *Trebouxia* (e.g., Mansournia et al., 2012; Dal Grande et al., 2014), Sanger sequencing of PCR amplicons has been shown to consistently identify the most abundant photobiont genotype (Paul et al., 2018). Furthermore, when multiple photobiont genotypes are present, they are generally closely related, belonging to the same species (Mansournia et al., 2012; Dal Grande et al., 2014), sister taxa (Casano et al., 2011), or at least the same group within *Trebouxia* (Paul et al., 2018).

For one specimen (Flakus 29597), Sanger sequencing recovered two different ITS sequences with the forward and reverse primers, corresponding to the photobionts I18 and S02. Repeated PCR and sequencing of this specimen generated the same result: two clean chromatograms with different sequences. The *rbc*L sequence obtained from this specimen was consistent with I18, so we used that photobiont in the figures and analyses, although S02 is also consistent with the ecology of this specimen. A technique such as fluorescence *in situ* hybridization (FISH) would be required to conclusively demonstrate that both photobionts occur in the same lichen thallus.

Specimens for which the photobiont ITS PCR was successful (as determined by a single clear band on an agarose gel) but sequencing failed because the chromatograms were unusable due to double peaks might plausibly represent cases where multiple algal species were present in the thallus. Likewise, cases where isolated double peaks occurred in otherwise high-quality chromatograms may indicate the presence of multiple algal haplotypes in a single thallus. Further investigation with next-generation sequencing would be required to verify these conjectures.

### Photobiont Switching Within a Mycobiont Species

Photobiont switching within a mycobiont species can occur at multiple phylogenetic levels: between photobiont genera (Ertz et al., 2018; Vančurová et al., 2018), between major clades within a photobiont genus (Blaha et al., 2006), between photobiont species within a subgeneric clade (Magain et al., 2017, 2018; Dal Grande et al., 2018), or between haplotypes within a photobiont species (Gasulla et al., 2020). We found no evidence of switching at the genus level, reaffirming that Lecanoraceae mycobionts are strict specialists on *Trebouxia* (Miadlikowska et al., 2006). Conversely, we found evidence for all three of the intrageneric types of switching, suggesting that the specificity of these mycobionts for particular algal species may be lower than commonly thought. It should be emphasized, however, that our sampling included few thalli from any one mycobiont species and is therefore not ideal for studying photobiont interactions within a mycobiont species. The conclusions drawn from the instances of switching we did observe should be considered preliminary and should be tested with a more focused sampling.

One of the mycobiont species we found with photobionts from two different *Trebouxia* clades was *Lecanora rupicola* (Figure 4). Blaha et al. (2006) sequenced the photobiont for specimens of *L. rupicola* from across Europe. They found that this species associated with diverse photobionts from three of the main clades of *Trebouxia*, including in some cases multiple photobiont species at a single site. The mycobiont was not sequenced in that study, so we do not know what role population structure or cryptic diversity may have played in the observed patterns, although the authors noted that there was no correlation between variation in secondary chemistry and photobiont identity.

All of the mycobiont species associated with photobionts from multiple *Trebouxia* clades occurred on siliceous rocks in open areas at mid-high to high elevation (Figure 4). The same was true for many of the mycobiont species that partnered with multiple photobionts from a single *Trebouxia* clade (Figure 4). This is consistent with previous work that has shown that mycobiont–photobiont associations in lichens are more specialized in warmer climates (Singh et al., 2017). One hypothesis to explain this pattern is that there are simply more photobiont species, and therefore greater opportunity for partner switching, at high elevation (Figure 6A). However, many of the instances of switching we observed were related to elevation change (Figure 5), lending support to the alternate hypothesis that mycobionts switch between locally-adapted photobionts along their range (Muggia et al., 2014; Dal Grande et al., 2018).

### Turnover of Mycobionts and Photobiont Communities

**FIGURE 7:**
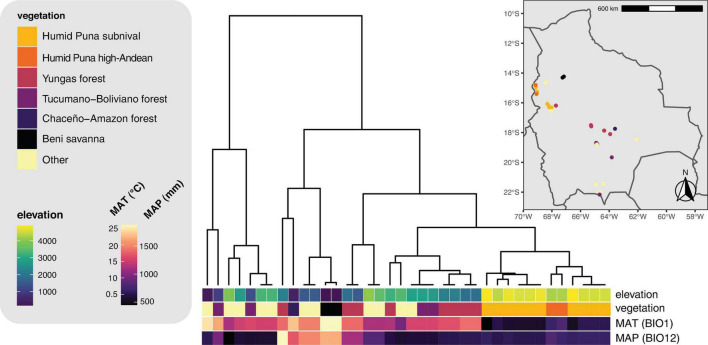
Hierarchical clustering dendrogram of 19 bioclimatic variables for the sampling localities. Sampling locations and vegetation types are displayed on the inset map. MAT, mean annual temperature (WorldClim variable BIO1). MAP, mean annual precipitation (WorldClim variable BIO12).

Our results add to a growing body of literature showing that elevation gradients have a substantial effect on the composition of lichen photobiont communities (Dal Grande et al., 2018; Devkota et al., 2019; Gasulla et al., 2020; Rolshausen et al., 2020; Wagner et al., 2020). This literature is part of a broader realization that microbial diversity is subject to some of the same biogeographic influences as macroscopic organisms (e.g., Nottingham et al., 2018; but see Bryant et al., 2008). For example, various taxa of free-living cyanobacteria and green algae in the soil may vary in their abundance along elevation gradients (Řeháková et al., 2011; Stewart et al., 2021). The elevation difference between the lowest and highest sites in our dataset—nearly 5,000 m, greater than any other study of this type—presents a unique opportunity to study mycobiont and photobiont turnover accompanying the transition between geographically close but ecologically distant biomes (Figure 7). In contrast to previous studies where turnover of mycobiont and photobiont communities occurred at different spatial scales (Dal Grande et al., 2018; Lu et al., 2018; Gasulla et al., 2020; Rolshausen et al., 2020), we observed turnover in Lecanoraceae communities and their associated *Trebouxia* photobionts at roughly equivalent rates (Figures 5, 6). However, because there are no mycobiont species that occur along the entire length of the gradient, we cannot tease apart the impact of substrate, climate, and mycobiont host on photobiont distribution to the degree possible in other studies (e.g., Dal Grande et al., 2018). For example, species of *Myriolecis* are almost always found on calcareous rock at high elevation with *Trebouxia* clade A.

Our findings on species richness and β-diversity along the elevation gradient are consistent with previously observed patterns and also suggest phenomena which should be investigated in a larger sampling replicated across multiple transects. The finding that, within each elevation bin, *Trebouxia* species were less diverse than Lecanoraceae (Figure 6A) recalls the conventional wisdom that natural communities contain more mycobiont species than photobiont species (e.g., Singh et al., 2019; Wagner et al., 2020). The one exception at 3,500 m, where the number of mycobiont and photobiont species are equal, corresponds to a poorly-sampled elevation band and may be an artifact (Figure 5).

Our results for β-diversity suggest an interesting phenomenon: Along an elevation gradient, species turnover occurs for both partners in a mutualistic interaction, but the elevation thresholds at which community composition shifts most dramatically are different for the two partners (Figure 6B). This pattern has not been seen in similar studies of smaller elevation gradients and fewer mycobiont taxa (e.g., Dal Grande et al., 2018). These thresholds may be equivalent to the “symbiont turnover zones” described by Rolshausen et al. (2020), but while those authors found a turnover zone for one partner, we show turnover zones for both partners and, crucially, these are at widely spaced elevations. No Lecanoraceae species are found both above and below 2,500 m, while no *Trebouxia* species are found on both sides of 3,500 m. The former elevation is the threshold at which rock begins to appear as a substrate for Lecanoraceae, and is also the transition point between aseasonal Yungas forest and seasonal Tucumano-Boliviano forest (Figures 4, 7). The latter elevation corresponds to the transition from *Trebouxia* clades C and I to *Trebouxia* clades A and S (Figure 5B); this is also the threshold at which open, rocky habitats fully replace forest vegetation (Figure 7). Perhaps not coincidentally, 3,500 m is also very close to a similar threshold for woody plant ranges in the Bolivian Andes (Tello et al., 2015).

These findings suggest two alternative hypotheses. One possibility is that mycobiont and photobiont ranges are responding to different biotic or abiotic factors (e.g., substrate versus temperature; rainfall versus the presence of pathogens). Another possibility is that both thresholds are a response to the same factor, but the two partners are responding differently. Lu et al. (2018) found that ranges of *Peltigera* species and *Nostoc* phylogroups did not have the same response to climate shifts along a latitudinal gradient, but their study was at an intra-biome scale and lacked the sharp community discontinuities we see here. While our sampling is not adequate to distinguish between these hypotheses, it provides guidance as to an ideal region and study design that would have the power to resolve this question.

### Phylogenetically Versus Ecologically Designed Sampling to Study Photobiont Communities

Kosecka et al. (2021b) examined the community of *Trebouxia* photobionts along a largely identical elevation gradient in Bolivia. That study included a four- to five-fold larger sample size from multiple families of *Trebouxia*-associated Lecanoromycetes, including some Lecanoraceae, but only dealt with the photobiont sequences from these specimens. The results of Kosecka et al. (2021b) in terms of *Trebouxia* diversity and distribution are largely consistent with our own findings, from the broad pattern of turnover among major clades of *Trebouxia* along the elevation gradient to many of the individual *Trebouxia* species found. Our study recovered several novel lineages not found by Kosecka et al. (2021b)—A53, C35, and C36; conversely, and commensurate with their larger sample size, Kosecka et al. (2021b) found several putative *Trebouxia* species which were missing from our sampling. Nevertheless, the close correspondence between the two studies suggests that dense sampling of a single mycobiont family may be sufficient to reveal regional patterns of biodiversity in lichenized *Trebouxia*. This stands in contrast to the situation with cyanolichens, where the *Nostoc* community will look very different if, for example, one samples from only Collemataceae, Peltigeraceae, or Lobariaceae in the same geographic area (Magain et al., 2018). Studies of *Trebouxia* photobionts from a single mycobiont clade are the norm in the literature, but it has not been clear whether their results can be generalized to represent the entire *Trebouxia* community of a region. Our results suggest that the answer may be yes, as long as the focal mycobiont clade occurs in a sufficiently diverse range of micro- and macrohabitats.

## Data Availability Statement

The datasets presented in this study can be found in online repositories. New sequence data generated for this study are available on GenBank with accession numbers OK665489–OK665671, OL603980–OL604141, OL625025–OL625113, and OL663852–OL663919. Other data are included in the online Supplementary Material for this article.

## Author Contributions

FL, JM, and LŚ contributed to the conception and design of the study and supervised Ph.D. students. LŚ and JM secured funding. AF and PR-F collected the specimens and ecological data in the field. EM and LŚ studied specimen morphology and delimited morphospecies. EM and EC prepared DNA extractions. IM designed primers for PCR and sequencing and generated the figures. EM, IM, and CP-DH collected the molecular data. IM and EM curated, validated, and analyzed the data and wrote the original draft of the manuscript. All authors participated in review and editing of the final manuscript and approved the submitted version.

## Conflict of Interest

The authors declare that the research was conducted in the absence of any commercial or financial relationships that could be construed as a potential conflict of interest.

## Publisher’s Note

All claims expressed in this article are solely those of the authors and do not necessarily represent those of their affiliated organizations, or those of the publisher, the editors and the reviewers. Any product that may be evaluated in this article, or claim that may be made by its manufacturer, is not guaranteed or endorsed by the publisher.

## Footnotes

1 https://eurofinsgenomics.eu/en/ecom/tools/pcr-primer-design/ (accessed June 24, 2021).

2 https://www.thermofisher.com/us/en/home/brands/thermo-scientific/molecular-biology/molecular-biology-learning-center/molecular-biologyresource-library/thermo-scientific-web-tools/multiple-primer-analyzer.html (accessed June 24, 2021).

3
https://www.geneious.com

4
https://mafft.cbrc.jp/alignment/server/

5
https://species.h-its.org/

6
https://decifr.hpc.ncsu.edu/index.php
