# Supplementary material for: Turnover of Lecanoroid Mycobionts and Their Trebouxia Photobionts Along an Elevation Gradient in Bolivia Highlights the Role of Environment in Structuring the Lichen Symbiosis
Source: Front Microbiol. 2021 Dec 20;12:774839. doi: 10.3389/fmicb.2021.774839 (PMC8721194; doi:10.3389/fmicb.2021.774839)
Supplement: Supplementary file 9 [file Data_Sheet_9.docx]

Supplementary Material

# Captions for Supplementary Data Sheets

Supplementary Data Sheet 1. Specimen data for samples sequenced in this study, including mycobiont species, *Trebouxia* species, collection locality data, and GenBank accession numbers for all newly obtained sequences.

Supplementary Data Sheet 2. GenBank accession numbers for reference taxa used in the phylogenetic analysis of Lecanoraceae.

Supplementary Data Sheet 3. Concatenated 6-locus alignment for Lecanoraceae. Loci are delimited as character sets and excluded regions are delimited as exclusion sets.

Supplementary Data Sheet 4. Concatenated ITS-*rbc*L alignment across the entire *Trebouxia* genus. Loci are delimited as character sets and excluded regions are delimited as exclusion sets.

Supplementary Data Sheet 5. Concatenated ITS-*rbc*L alignment for *Trebouxia* clade A. Loci are delimited as character sets and excluded regions are delimited as exclusion sets.

Supplementary Data Sheet 6. Concatenated ITS-*rbc*L alignment for *Trebouxia* clade C. Loci are delimited as character sets and excluded regions are delimited as exclusion sets.

Supplementary Data Sheet 7. Concatenated ITS-*rbc*L alignment for *Trebouxia* clade I. Loci are delimited as character sets and excluded regions are delimited as exclusion sets.

Supplementary Data Sheet 8. Concatenated ITS-*rbc*L alignment for *Trebouxia* clade S. Loci are delimited as character sets and excluded regions are delimited as exclusion sets.

# Captions for Supplementary Figures

Supplementary Figure 1. ITS-*rbcL* tree of *Trebouxia*. Bold tip names indicate specimens sequenced in this study. Major clades are highlighted with the color scheme used throughout this paper. Support values are UFboot2 with 5000 replicates. Scale indicates substitutions per site.

Supplementary Figure 2. ITS-*rbcL* tree of *Trebouxia* clade A. Bold tip names indicate specimens sequenced in this study. Putative species present in our sampling are annotated. Support values are UFboot2 with 5000 replicates. Scale indicates substitutions per site.

Supplementary Figure 3. ITS-*rbcL* tree of *Trebouxia* clade C. Bold tip names indicate specimens sequenced in this study. Putative species present in our sampling are annotated. Support values are UFboot2 with 5000 replicates. Scale indicates substitutions per site.

Supplementary Figure 4. ITS-*rbcL* tree of *Trebouxia* clade I. Bold tip names indicate specimens sequenced in this study. Putative species present in our sampling are annotated. Support values are UFboot2 with 5000 replicates. Scale indicates substitutions per site.

Supplementary Figure 5. ITS-*rbcL* tree of *Trebouxia* clade S. Bold tip names indicate specimens sequenced in this study. Putative species present in our sampling are annotated. Support values are UFboot2 with 5000 replicates. Scale indicates substitutions per site.

Supplementary Figure 6. Single-locus tree for Lecanoraceae ITS. Maximum-likelihood tree inferred with the K2P+R3 substitution model. Support values are UFboot2 with 5000 replicates. Bipartitions with bootstrap support ≥95 are indicated with bold branches. Bold tip names indicate specimens sequenced in this study. Scale indicates substitutions per site.

Supplementary Figure 7. Single-locus tree for Lecanoraceae nrLSU. Maximum-likelihood tree inferred with the TN+F+R3 substitution model. Support values are UFboot2 with 5000 replicates. Bipartitions with bootstrap support ≥95 are indicated with bold branches. Bold tip names indicate specimens sequenced in this study. Scale indicates substitutions per site.

Supplementary Figure 8. Single-locus tree for Lecanoraceae mtSSU. Maximum-likelihood tree inferred with the HKY+F+R3 substitution model. Support values are UFboot2 with 5000 replicates. Bipartitions with bootstrap support ≥95 are indicated with bold branches. Bold tip names indicate specimens sequenced in this study. Scale indicates substitutions per site.

Supplementary Figure 9. Single-locus tree for Lecanoraceae *RPB1*. Maximum-likelihood tree inferred with the SYM+I+G4 substitution model. Support values are UFboot2 with 5000 replicates. Bipartitions with bootstrap support ≥95 are indicated with bold branches. Bold tip names indicate specimens sequenced in this study. Scale indicates substitutions per site.

Supplementary Figure 10. Single-locus tree for Lecanoraceae *RPB2*. Maximum-likelihood tree inferred with the SYM+R5 substitution model. Support values are UFboot2 with 5000 replicates. Bipartitions with bootstrap support ≥95 are indicated with bold branches. Bold tip names indicate specimens sequenced in this study. Scale indicates substitutions per site.

Supplementary Figure 11. Single-locus tree for Lecanoraceae *MCM7*. Maximum-likelihood tree inferred with the TIM+F+I+G4 substitution model. Support values are UFboot2 with 5000 replicates. Bipartitions with bootstrap support ≥95 are indicated with bold branches. Bold tip names indicate specimens sequenced in this study. Scale indicates substitutions per site.

Supplementary Figure 12. Six-locus tree of Lecanoraceae. Bold tip names indicate specimens sequenced in this study. Genera and other major clades are annotated. Support values are UFboot2 with 5000 replicates. Scale indicates substitutions per site.
