# Supplementary figures and images for: Turnover of Lecanoroid Mycobionts and Their Trebouxia Photobionts Along an Elevation Gradient in Bolivia Highlights the Role of Environment in Structuring the Lichen Symbiosis
Source: Front Microbiol. 2021 Dec 20;12:774839. doi: 10.3389/fmicb.2021.774839 (PMC8721194; doi:10.3389/fmicb.2021.774839)

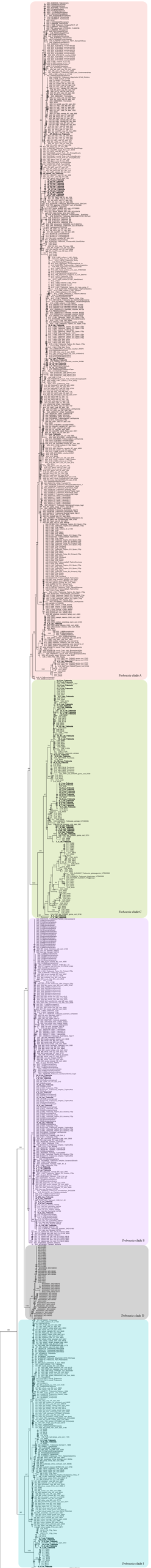

Supplement: Supplementary file 10 [file Image_1.pdf]

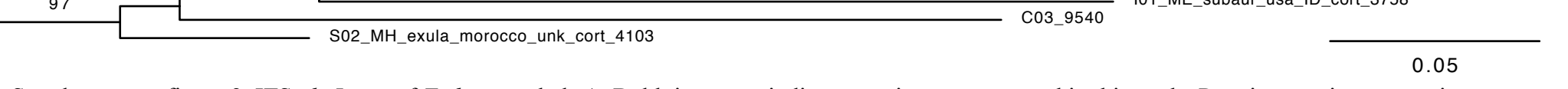

Supplement: Supplementary file 11 [file Image_2.pdf]
