# Supplementary material for: Turnover of Lecanoroid Mycobionts and Their Trebouxia Photobionts Along an Elevation Gradient in Bolivia Highlights the Role of Environment in Structuring the Lichen Symbiosis
Source: Front Microbiol. 2021 Dec 20;12:774839. doi: 10.3389/fmicb.2021.774839 (PMC8721194; doi:10.3389/fmicb.2021.774839)
Supplement: Supplementary file 14 [file Image_5.pdf]

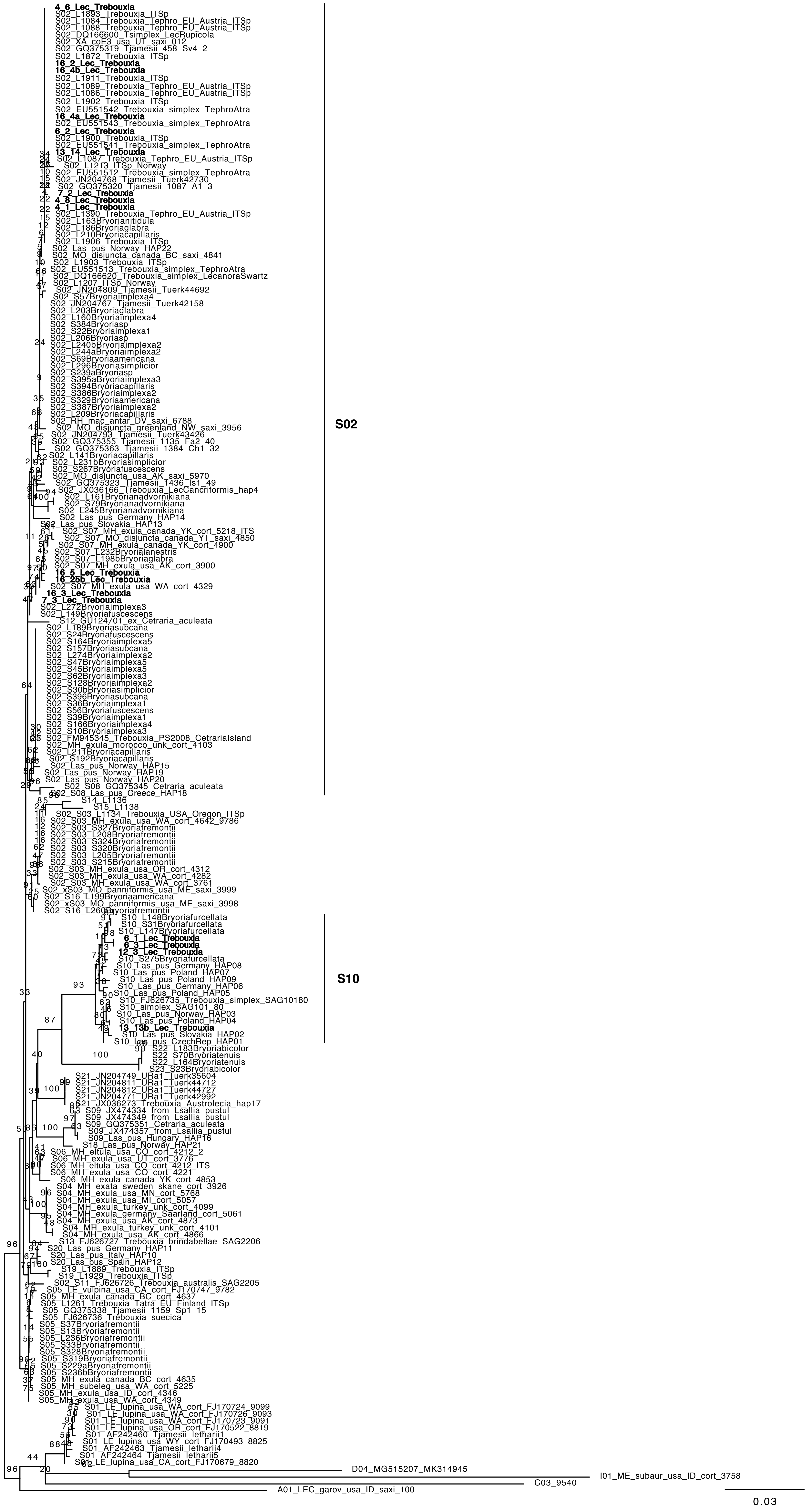

Supplementary figure 5. ITS-*rbcL* tree of *Trebouxia* clade S. Bold tip names indicate specimens sequenced in this study. Putative species present in our sampling are annotated. Support values are UFboot2 with 5000 replicates. Scale indicates substitutions per site.
