# Supplementary material for: Turnover of Lecanoroid Mycobionts and Their Trebouxia Photobionts Along an Elevation Gradient in Bolivia Highlights the Role of Environment in Structuring the Lichen Symbiosis
Source: Front Microbiol. 2021 Dec 20;12:774839. doi: 10.3389/fmicb.2021.774839 (PMC8721194; doi:10.3389/fmicb.2021.774839)
Supplement: Supplementary file 18 [file Image_9.pdf]

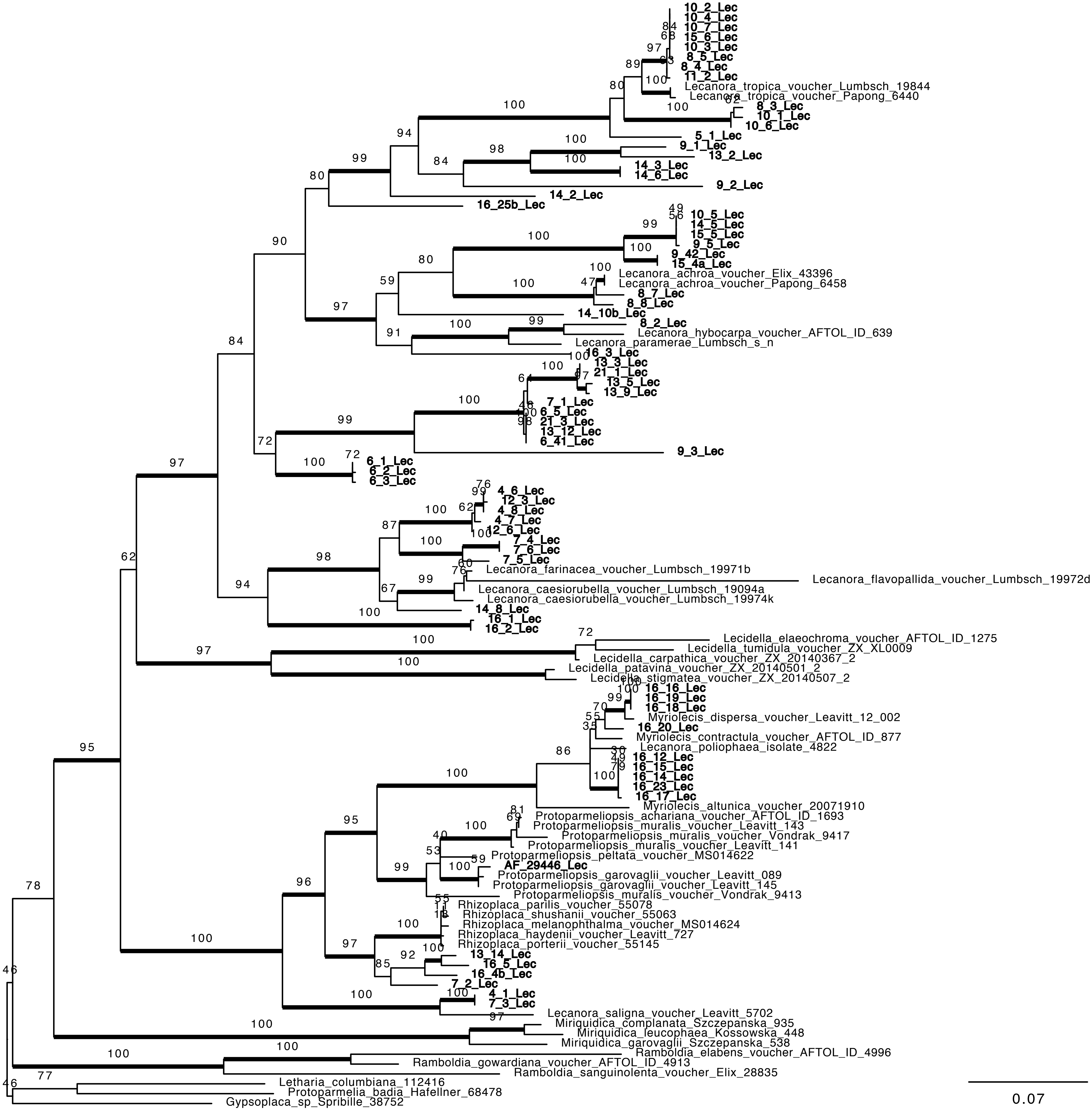

Supplementary figure 9. Single-locus tree for Lecanoraceae *RPB1*. Maximum-likelihood tree inferred with the SYM+I+G4 substitution model. Support values are UFboot2 with 5000 replicates. Bipartitions with bootstrap support ≥95 are indicated with bold branches. Bold tip names indicate specimens sequenced in this study.Scale indicates substitutions per site.
