# Supplementary material for: Turnover of Lecanoroid Mycobionts and Their Trebouxia Photobionts Along an Elevation Gradient in Bolivia Highlights the Role of Environment in Structuring the Lichen Symbiosis
Source: Front Microbiol. 2021 Dec 20;12:774839. doi: 10.3389/fmicb.2021.774839 (PMC8721194; doi:10.3389/fmicb.2021.774839)
Supplement: Supplementary file 21 [file Image_12.pdf]

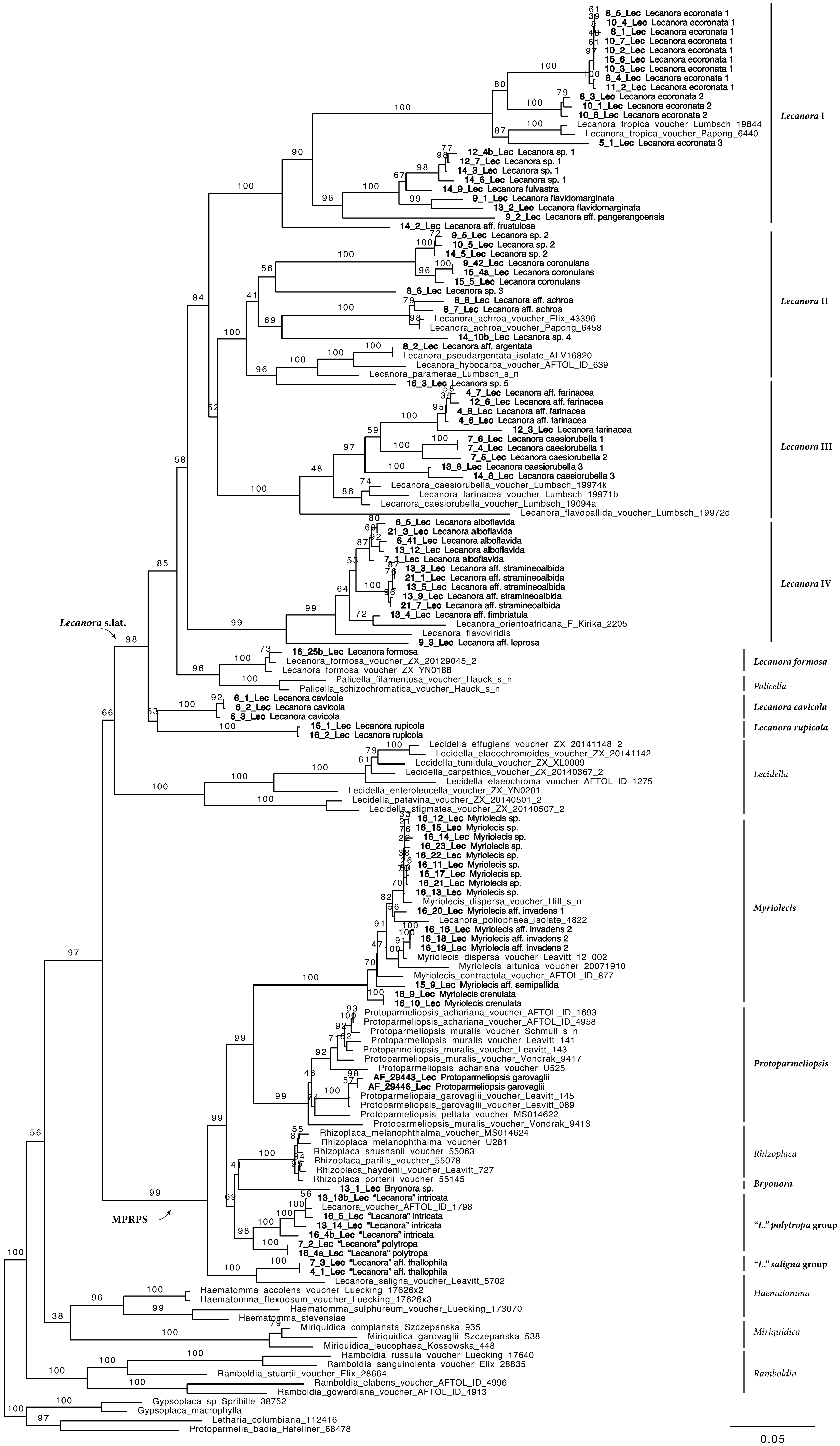

Supplementary figure 12. Six-locus tree of Lecanoraceae. Bold tip names indicate specimens sequenced in this study. Genera and other major clades are annotated. Support values are UFboot2 with 5000 replicates. Scale indicates substitutions per site.
